# Therapeutic value of homeoprotein signaling pathways

**DOI:** 10.3389/fnins.2024.1359523

**Published:** 2024-03-14

**Authors:** Ariel A. Di Nardo, Alain Prochiantz

**Affiliations:** Center for Interdisciplinary Research in Biology (CIRB), College de France, CNRS, INSERM, Université PSL, Paris, France

**Keywords:** homeoproteins, development, neurological diseases, psychiatric diseases, protein therapy

## Abstract

Cell signaling based on homeoprotein transfer is a pathway with developmental and physiological functions. For a few transcription factors of this family, primarily ENGRAILED1, ENGRAILED2 and OTX2, their physiological functions have led to therapeutic strategies in animal models of human diseases, including Parkinson’s disease, amyotrophic lateral sclerosis, amblyopia and anxiety-related disorders. In mesencephalic dopaminergic neurons which degenerate in Parkinson’s disease, ENGRAILED1/2 have cell autonomous activities, but their transducing properties enables their use as therapeutic proteins. In contrast, in spinal alpha-motoneurons, which are lost in amyotrophic lateral sclerosis, ENGRAILED1 is supplied by V1 interneurons. Thus, its use as a therapeutic protein to protect alpha-motoneurons against degeneration mimics its normal non-cell autonomous neurotrophic activity. OTX2, synthesized and secreted by the choroid plexus, is transferred to parvalbumin interneurons and exerts regulatory functions controlling cerebral cortex plasticity. Understanding the latter OTX2 function has led to strategies for manipulating visual acuity and anxiety-like behavior in adult mice. In this review, we describe these cases and what is known about the involved molecular mechanisms. Because the transduction sequences are conserved in most of the few hundred homeoproteins, we argue how this family of molecules constitutes an important reservoir of physiological knowledge, with potential consequences in the search for new therapeutic strategies.

## Introduction

### The discovery of homeoprotein signaling

Transcription factor participation in signal transduction is normally cell autonomous. Homeoprotein (HP) transcription factors, discovered on the basis of their developmental functions but expressed throughout adulthood, provide an exception to the rule. Direct non-cell autonomous signaling by cell-to-cell HP transfer was first discovered in plants where KNOTTED1 travels through specific cell bridging structures called plasmodesmata (Lucas et al., 1995; Kim et al., 2002; Ruiz-Medrano et al., 2004; Bolduc et al., 2008). In animals, a first step in the discovery of HP transfer was the observation that their DNA-binding domain, or homeodomain (HD), is internalized by live cells and directly addressed to the cytoplasm and nucleus (Joliot et al., 1991). This finding was rapidly followed by the demonstration that full-length HPs are internalized and secreted through non-conventional mechanisms (Prochiantz and Di Nardo, 2015; Di Nardo et al., 2018, 2020). HP secretion and internalization domains are part of the highly conserved HD explaining why most of the 160 tested HPs can transfer (Lee et al., 2019). Despite this high number and the likeliness that this property is shared by the approximately 300 members of the HP family, the developmental and physiological functions associated with HP transfer have been studied for only a few of them, including ENGRAILED (EN), PAX6, VAX1 and OTX2. Before illustrating with EN1 and OTX2 how this novel signaling pathway is conducive to new therapeutic strategies, we will describe established HP signaling functions and the mechanisms involved.

### Summary of established HP signaling functions in animals

The main established functions are summarized in Table 1. During development, PAX6 signaling acts on cell migration, as shown for Cajal–Retzius cells in the embryonic mouse cerebral cortex (Kaddour et al., 2019), and for oligodendrocyte precursor cells (OPCs) in the chick spinal cord (Di Lullo et al., 2011). Still during development, EN signaling regulates anterior cross vein (ACV) formation in the Drosophila wing disk (Layalle et al., 2011), eye anlagen development and midbrain patterning in the zebrafish (Lesaffre et al., 2007; Rampon et al., 2015; Amblard et al., 2020b), and retinal ganglion cell (RGC) axon guidance and synaptic stability in the chick and frog (Brunet et al., 2005; Wizenmann et al., 2009; Yoon et al., 2012). In RGC growth cones, EN1 and EN2 (together EN1/2) activity involves the regulation of local mRNA translation (Brunet et al., 2005, 2007; Wizenmann et al., 2009). Some of these mRNAs encode mitochondrial complex I proteins and their EN1/2-induced translation results in transient ATP synthesis (Stettler et al., 2012). Also related to growth cone behavior, VAX1 was shown to regulate RGC decussation at the level of the optic chiasma (Kim et al., 2014; Min et al., 2023). In the adult, EN1 secreted by V1 interneurons in the mouse spinal cord is captured by motoneurons (MNs), and blocking this transfer induces αMN retrograde degeneration (Lebœuf et al., 2023). In the juvenile and adult mouse, OTX2 signaling regulates the opening and closure of cerebral cortex critical periods (CPs) of plasticity in the visual, auditory, and medial prefrontal cortices (Sugiyama et al., 2008). This regulation involves the secretion of OTX2 by the choroid plexus and its specific capture by parvalbumin interneurons (PV cells) localized in layer IV of the cerebral cortex (Beurdeley et al., 2012; Spatazza et al., 2013; Bernard et al., 2016). OTX2 internalization by PV cells induces their maturation and a shift in the excitatory/inhibitory (E/I) balance toward inhibition leading to heightened neural circuit plasticity (Sugiyama et al., 2008). In the visual system, blocking OTX2 signaling in the mouse retina within a week after eye opening delays CP onset (Sugiyama et al., 2008).

**Table 1:** Main identified homeoprotein developmental and adult signaling functions.

HP	Model	Embryo	Juvenile	Adult
KN1	Plant shoot meristem			
PAX6	Zebrafish	Eye anlagen		
	Chick	OPC migration		
	Mouse	CR cell migration		
EN	Fly	Wing disk ACV		
	Zebrafish	Midbrain Patterning		
EN1/2	Xenopus	RGC axon guidance	Synapse stabilization	
	Chick	RGC axon guidance		
	Mouse	RGC axon guidance		⍺MN survival
VAX1	Mouse	RGC axon decussation		
OTX2	Xenopus	RGC axon guidance		
	Mouse		Critical Period timing	Cortical plasticity

This table illustrates for only a few homeoproteins the variety of species and developmental or adult processes in which their direct signaling properties have been demonstrated. ACV, anterior cross vein; CR, Cajal–Retzius; HP, homeoprotein; OPC, oligodendrocyte precursor cell; RGC, retinal ganglion cell. See references in text.

## Mechanisms of intercellular transfer and co-signaling

### Rapid insight into HP transfer mechanisms and specificity

Signaling requires HP secretion and internalization. Both processes involve specific domains within the HD and are unconventional in the sense that secretion is not through the ER-Golgi pathway while internalization does not require endocytosis and instead involves crossing the plasma membrane with direct access to the cytoplasm and nucleus. Despite years of research by several laboratories, including chemists and cell biologists, the internalization and secretion processes are not yet fully understood. Although transfer mechanisms were mainly studied with EN2 and OTX2, domain conservation suggests most HPs use similar strategies. EN2 secretion and internalization involve an interaction with phosphatidylinositol (4,5)-biphosphate (PIP2), and a recent report suggests that OTX2 secretion involves association with nuclear membrane buds and lysosomes (Amblard et al., 2020a; Park et al., 2023). From a therapeutic perspective, two main points are of particular interest. The first one is direct access to the cytoplasm, first demonstrated for the HP internalization domain defined by the third helix of the HD and known as *Penetratin*. Direct access may involve the formation of inverted micelles (Derossi et al., 1994, 1996, 1998; Berlose et al., 1996) and/or membrane hyperpolarization (Trofimenko et al., 2021). A second point is the specificity of cell targeting by HPs. In the case of EN1 and OTX2, the specific recognition of spinal αMNs and PV cells, respectively, is due to the interaction between glycosaminoglycans (GAGs) present at the cell surface and a GAG-binding motif overlapping with the HD first helix (Beurdeley et al., 2012; Lebœuf et al., 2023). Although two HPs are not sufficient to validate a “sugar code” hypothesis for *in vivo* specific targeting, it is of note that GAG binding domains are present at a similar sequence position in many HPs (Prochiantz and Di Nardo, 2015).

### Co-signaling

An interesting aspect of HP signaling is that it can work synergistically with classical signaling. The best example is EN2 signaling that provokes the *in vitro* collapse of temporal retina RGC growth cones, similar to aggregated EphrinA5. In an experiment where temporal cones were given the choice to navigate on naive or EphrinA5-coated stripes, EphrinA5 avoidance was clear at 0.5 μg/mL but not at a 0.1 μg/mL, unless 75 nM EN2 was added to the culture medium (Wizenmann et al., 2009). The same cooperation was replicated by directly monitoring growth cone collapse, revealing that cooperative EN2/EphrinA5 signaling requires EN2 internalization and is mediated by local translation (Wizenmann et al., 2009). In fact, further experiments demonstrated that local translation is followed by a burst of ATP synthesis and that adenosine produced by extracellular ATP degradation activates Adenosine receptor A1, providing an intermediate step in EN2-potentiated EphrinA5 signaling (Stettler et al., 2012). Two other examples, not developed here, include the interaction between PAX6 and netrin signaling for OPC migration (Di Lullo et al., 2011), and that of EN and Decapentaplegic (DPP) signaling in the activation of Mothers Against DPP, resulting in ACV formation in the fly wing imaginal disc (Layalle et al., 2011). This concept is important to bear in mind for HP signaling mechanisms, as the exact active morphogen concentrations *in vivo* are unknown and HP transfer might thus be a co-signaling partner in several developmental and physiological situations.

## Homeoprotein therapeutic activities in animal models of human diseases

**Figure 1:**
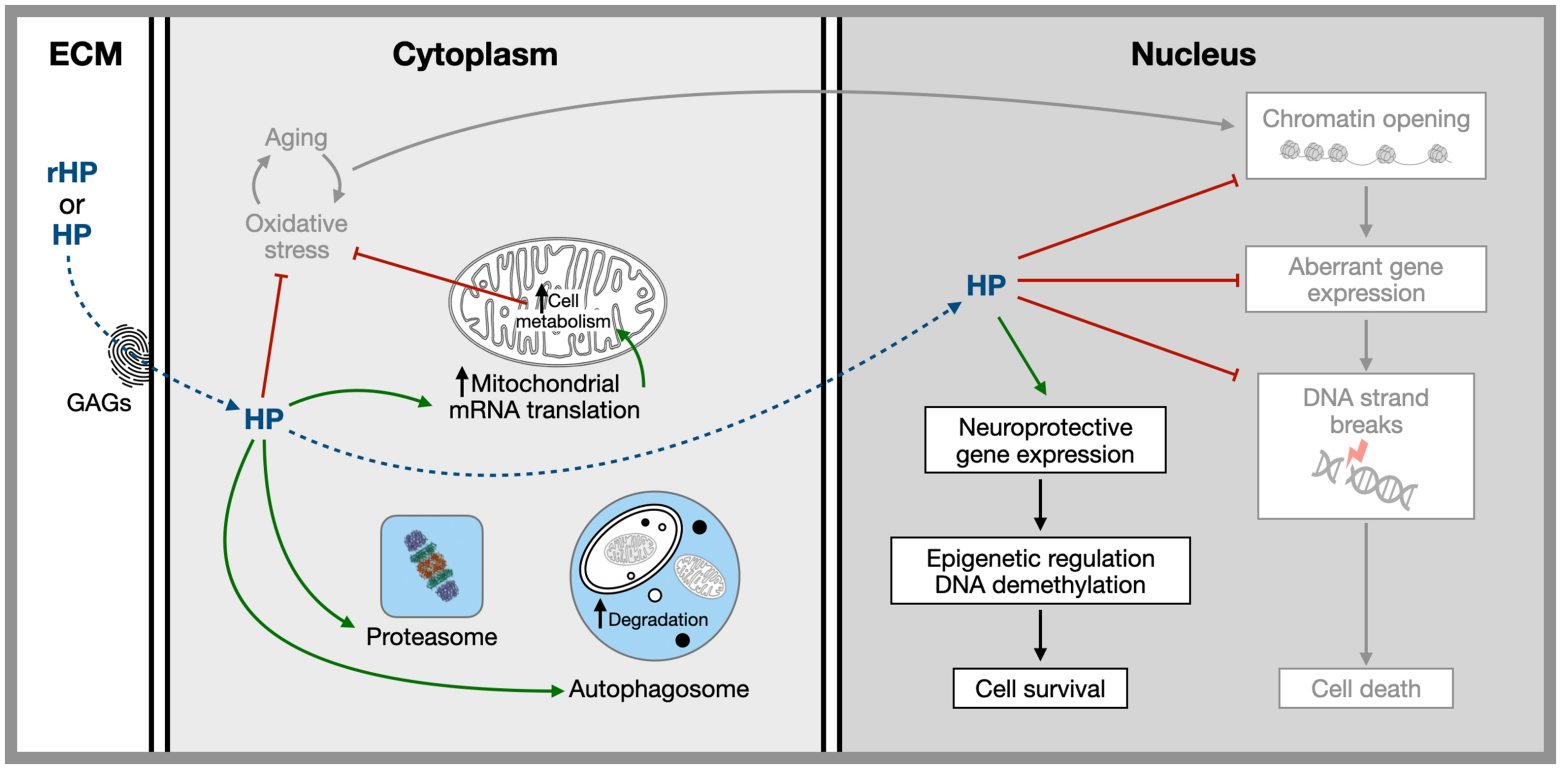
Hypothetical sites and mechanisms of homeoprotein non-cell autonomous activities based on the study of EN1, EN2, and OTX2. Homeoproteins secreted by physiological sources or injected *in vivo* gain access to specific target cells thanks to a glycosaminoglycan “fingerprint” recognition code. Once internalized they have both cytoplasmic and nuclear activities related to several aging hallmarks including, mitochondrial activity, protection against oxidative stress, regulation of proteostasis, the regulation of expression of neuroprotective genes and the protection of the chromatin landscape allowing for the repression within the heterochromatin of illegitimate genes presenting neuronal harming properties. References in the text.

The use of HPs and HP-derived tools in the regulation of physiological functions is recalled in Table 2. Potential HP-associated therapeutic pathways are summarized in Figure 1. They include local translation, transcription regulation, and chromatin organization with an impact on genome stability.

**Table 2:** Main utilizations of homeoproteins, homeoprotein-derived peptides and homeoprotein antagonists in the regulation of physiological functions.

Tool	Model	Target
*Penetratin*-LINE1-siRNA	Mouse	Parkinson’s disease
EN1/2	Mouse	Parkinson’s disease
	Mouse	Amyotrophic lateral sclerosis
	Macaque	Parkinson’s disease
OTX2	Mouse	Glaucoma
OTX2 antagonists	Mouse	Amblyopia
	Mouse	Anxiety-like behavior

Penetratin, a cell-penetrating peptide corresponding to the third helix of the Antennapedia homeodomain has been widely used for the in vitro and in vivo internalization of a large number of peptides, phospho-peptides, and oligonucleotides. Here is recalled the in vivo use of Penetratin to internalize in midbrain neurons a siRNA targeting the Orf2p sequence present in the LINE-1 polycistronic mRNA. OTX2 antagonists used are single-chain anti-OTX2 antibodies and an OTX2-derived peptide competing for OTX2 binding to PV-cell extracellular matrix and its ensuing internalization. See references in text.

### EN1/2 as a therapeutic protein in animal models of Parkinson’s disease

Mesencephalic dopaminergic (mDA) neurons that innervate the striatum and degenerate in Parkinson’s disease (PD) express both EN1 and EN2. In the Swiss genetic background, *En1* heterozygote (*En1*-Het) mice experience progressive mDA neuron retrograde degeneration and develop motor and non-motor PD-like symptoms (Sonnier et al., 2007; Alvarez-Fischer et al., 2011). Changes in epigenetic mark distribution and intensity, for marks such as H3K9me27, H3K9me3, Nucleolin, and LaminB2, are observed specifically in *En1*-Het mice mDA neurons, together with an increase in the number of γH2AX foci (DNA-breaks) and the expression of LINE-1A and LINE-1Tf/Gf retrotransposons (Rekaik et al., 2015; Blaudin de Thé et al., 2018). In keeping the observation that mDA neurons from *En1*-Het mice are more sensitive to oxidative stress, the internalization of recombinant EN1/2 by mDA neuron rescues them from oxidative stress induced either by a 6-OHDA stereotaxic injection in the mouse or by slow MPTP exposure in the non-human primate (Rekaik et al., 2015; Thomasson et al., 2019). EN1/2 injection, and internalization by mouse mDA neurons, 30 min after 6-OHDA administration, rescues the cells from degeneration and returns all nuclear marks back to normal (Rekaik et al., 2015). The hypothesis that a mechanism of EN1/2 “therapeutic activity” involves LINE-1A is supported by the finding that EN2 directly represses LINE-1A expression and binds to its promoter (Blaudin de Thé et al., 2018). Further evidence that substantiates the LINE-1A (and possibly Tf/Gf) hypothesis includes the protection against 6-OHDA by stavudine, a reverse-transcriptase inhibitor and siRNAs directed against LINE-1A ORF2 protein (Blaudin de Thé et al., 2018), and the protective activity of PIWIL1 protein (binds and inactivates LINE-1 transcripts) when overexpressed in the midbrain of *En1*-Het mice. These experiments suggest that in *En1*-Het or in WT mice exposed to oxidative stress, a loss of heterochromatin allows for toxic LINE-1 overexpression that can be repressed by EN1/2 gain of function, both directly at a transcriptional level and indirectly through heterochromatin restoration (Blaudin de Thé et al., 2018). It is of note that OTX2 exerts a similar protective activity on midbrain dopaminergic neurons and RGCs in a mouse glaucoma model (Torero-Ibad et al., 2011; Rekaik et al., 2015).

### Neurotrophic protective activity of non-cell autonomous ENGRAILED1 for spinal cord alpha-motoneurons

In the ventral spinal cord, V1 interneurons, including Renshaw cells, express EN1 while the *En1* locus is not active in αMNs (Wenner and O’Donovan, 1999; Sapir, 2004; Lebœuf et al., 2023). These large MNs receive synaptic input from V1 interneurons and capture secreted EN1 protein. When this transfer is blocked by the local expression of a secreted EN1-specific single-chain antibody (scFv-EN1), αMN retrograde degeneration is induced and muscular strength is partially lost (Lebœuf et al., 2023). A similar degenerative phenotype is observed in the Swiss *En1*-Het mouse (Lebœuf et al., 2023). Human recombinant EN1 (hEN1) injected intrathecally at lumbar 5 (L5) in mice gains access to the spinal cord parenchyma and is specifically captured by αMNs and γMNs, but not by any other cell type (Lebœuf et al., 2023). This specificity is dependent on a GAG-binding domain upstream of the HD, as was observed for the internalization of OTX2 by PV cells. A single 1 μg injection of hEN1 is sufficient to block αMN degeneration and restore endplate innervation with full neuromuscular strength for 3 months (Lebœuf et al., 2023). This long-lasting effect strongly suggests that EN1 activity engages epigenetic mechanisms, highly reminiscent of its activity in mDA neurons. Based on this similarity, a bioinformatic study was undertaken to identify genes expressed in human MNs that are differentially expressed in the Swiss *En1*-Het mouse substantia nigra and interact with one of the 4 main genes mutated in ALS familial forms (*SOD1*, *FUS*, *TARDBP-43* and *C9orf72*; Rekaik et al., 2015; Lebœuf et al., 2023). This approach generated a list of 20 genes, including *p62/SQSTM1* which was the only gene to interact with the 4 ALS genes (Lebœuf et al., 2023). *p62/SQSTMI* is mutated in some familial forms of ALS (Fecto et al., 2011; Shimizu et al., 2013; Hadano et al., 2016; Doherty and Baehrecke, 2018; Yilmaz et al., 2019; Foster and Rea, 2020) and encodes an autophagy protein that is considered a marker of aging (Hensley and Harris-White, 2015; Menzies et al., 2015; Tai et al., 2016; Leidal et al., 2018). p62/SQSTM1 expression increases with age in αMNs of WT mice and is increased in *En1*-Het mice or when EN1 transfer into αMNs is antagonized in WT mice (Lebœuf et al., 2023). In contrast, its expression is down-regulated following EN1 treatment of *En1*-Het mice (Lebœuf et al., 2023). Taken together, these results suggest that αMNs show accelerated aging in *En1*-Het mice and that EN1 is a “therapeutic” anti-aging protein working at an epigenetic level.

### The regulation of cerebral plasticity by OTX2 and its therapeutic outcomes

The cerebral cortex adapts to the surrounding environment during CPs of heightened plasticity that allow neural circuits to be remodeled by experience (Hensch, 2005). These CPs take place postnatally in different brain regions and involve many functions: visual, auditory, sensory-motor, linguistic, social, cognitive, etc. (Reh et al., 2020). Since the seminal studies of Hubel and Wiesel on binocular vision (Hubel and Wiesel, 1965, 1970; Wiesel and Hubel, 1965a,b; Wiesel, 1982), the CP for ocular dominance (OD) plasticity has garnered much attention. In the mouse, this CP opens at postnatal day 20 (P20), peaks around P28, and closes by P40, paralleling progressive PV cell maturation in response to OTX2 capture, which is mediated by specific binding to GAGs present within condensed extracellular matrix perineuronal nets (PNNs) that form around PV cells (Sugiyama et al., 2008; Beurdeley et al., 2012; Miyata et al., 2012). This capture of OTX2 is also progressive, with OTX2 levels being undetectable prior to CP onset, and then increasing in parallel with PNN levels (Sugiyama et al., 2008; Lee et al., 2017). OTX2 has a precise role in controlling CP timing. A gain of function of OTX2 at P17 results in peak plasticity at P20 and CP closure at P25, thus accelerating the entire maturation process (Sugiyama et al., 2008). Conversely, decreased OTX2 import into PV cells delays CP opening in the visual, auditory, and medial prefrontal cortices (Bernard et al., 2016; Lee et al., 2017). At the epigenetic level, OTX2 directly and rapidly upregulates *Gadd45ß* at CP onset, leading to changes in the pattern of CpG methylations that can impact transcription and chromatin structure (Apulei et al., 2018). OTX2 transfer from choroid plexus to PV cells is maintained throughout life, with maximal steady-state levels in the adult cortex (Spatazza et al., 2013). Long-term closing of one eye during CP, but not before or after, induces experimental amblyopia in the mouse, a condition also encountered in humans with juvenile monocular defects, such as strabismus or cataract, if not treated before 8 years of age (OD CP closure). Interestingly, transiently reopening plasticity by pharmacological OTX2 reduction in the adult cures experimental amblyopia in the mouse (Spatazza et al., 2013; Bernard et al., 2016; Apulei et al., 2018). Since OTX2 is captured by PV cells throughout the cortex (Spatazza et al., 2013), restoration of binocular vision in the amblyopic mouse is an example of a therapeutic protocol that may be of value for other neurodevelopmental diseases. Consistent with this idea, delaying OTX2 import interferes with the development of auditory tonotopic maps and mood-related behaviors (Lee et al., 2017). Strikingly, anxiety-like behaviors are attenuated in the *Otx2*-Het mouse and can be returned to normal in the adult by overexpressing OTX2 in the choroid plexus (Vincent et al., 2021). Conversely, reducing OTX2 levels in the cerebrospinal fluid installs a hypoanxious-like behavior implicating medial prefrontal cortex PV cells in the adult mouse (Vincent et al., 2021).

## Concluding remarks on homeoproteins as time-controlling agents

Molecular studies in PD animal models, and the long-lasting activity of a single EN1 injection on αMN survival and activity, strongly suggest that EN1/2 exert important functions at an epigenetic level. This hypothesis has weight in the context of neurodegenerative diseases for which age is a major risk factor, even in familial forms provoked by mutations that remain silent for several years. The primary hallmarks of aging include genomic instability, epigenetic alterations, loss of proteostasis, disabled macro-autophagy, and telomere attrition (López-Otín et al., 2023). In neurons, telomere attrition is not operational but it is striking that in EN1/2 protective activities are associated with LINE-1 repression, the restoration of most epigenetic marks, and the restoration of autophagy by p62/SQSTM1 regulation. Given that chromatin modifications, loss of autophagy and the upregulation of mobile genetic elements of the LINE family are associated with aging (Laurent et al., 2010; Maxwell et al., 2011; Li et al., 2013; Meter et al., 2014; Krug et al., 2017; Simon et al., 2018, 2019; Valle et al., 2022), EN1/2 has potential as an anti-aging and even a reverse-aging therapeutic protein for mouse mDA neurons and αMNs. But timing does not only implicate aging, as illustrated by the importance for proper synchrony between circuit refinement and environmental information in postnatal learning. This need is well illustrated by how the exact timing of CP windows is essential for physiological alignment between environmental inputs and intrinsic programs of circuit maturation. This makes OTX2 transfer a key factor in determining when functional plasticity opens and closes during postnatal development, with the kinetics and epigenetic impact of OTX2 accumulation in PV cells providing temporal control of neural circuit maturation to define CP timing. In this context, the fact that decreasing OTX2 levels in PV cells reopens plasticity after CP closure suggests that manipulating the OTX2 pathway can be used to “reverse” cortical aging and phenocopy juvenile properties in the adult. It will thus be of high interest to evaluate if such time-controlling functions identified for EN1 and OTX2 are valid for the other HPs expressed in neuronal populations affected in several neurological and psychiatric pathologies.

## Author contributions

AD: Writing – original draft, Writing – review & editing. AP: Writing – original draft, Writing – review & editing.
